# Enhancing Hydrogen Evolution Activity of Au(111) in Alkaline Media through Molecular Engineering of a 2D Polymer

**DOI:** 10.1002/anie.201915855

**Published:** 2020-03-18

**Authors:** Patrick Alexa, Juan Manuel Lombardi, Paula Abufager, Heriberto Fabio Busnengo, Doris Grumelli, Vijay S. Vyas, Frederik Haase, Bettina V. Lotsch, Rico Gutzler, Klaus Kern

**Affiliations:** ^1^ Max Planck Institute for Solid State Research Heisenbergstrasse 1 70569 Stuttgart Germany; ^2^ Instituto de Física Rosario and Universidad Nacional de Rosario CONICET-UNR S2000EZO Rosario Argentina; ^3^ Instituto de Investigaciones Fisicoquímicas Teóricas y Aplicadas (INIFTA) Facultad de Ciencias Exactas Universidad Nacional de La Plata, CONICET 1900 La Plata Argentina; ^4^ Department of Chemistry University of Munich (LMU) Butenandtstrasse 5–13 81377 München Germany; ^5^ Institut de Physique École Polytechnique Fédérale de Lausanne (EPFL) 1015 Lausanne Switzerland; ^6^ Present address: Department of Chemistry Marquette University Milwaukee WI 53233 USA; ^7^ Present address: Institute for Integrated Cell-Material Sciences (WPI-iCeMS) Kyoto University, iCeMS Research Bldg, Yoshida, Sakyo-ku Kyoto 606-8501 Japan

**Keywords:** density functional theory, hybrid catalyst, hydrogen evolution reaction, polymers, surface chemistry

## Abstract

The electrochemical splitting of water holds promise for the storage of energy produced intermittently by renewable energy sources. The evolution of hydrogen currently relies on the use of platinum as a catalyst—which is scarce and expensive—and ongoing research is focused towards finding cheaper alternatives. In this context, 2D polymers grown as single layers on surfaces have emerged as porous materials with tunable chemical and electronic structures that can be used for improving the catalytic activity of metal surfaces. Here, we use designed organic molecules to fabricate covalent 2D architectures by an Ullmann‐type coupling reaction on Au(111). The polymer‐patterned gold electrode exhibits a hydrogen evolution reaction activity up to three times higher than that of bare gold. Through rational design of the polymer on the molecular level we engineered hydrogen evolution activity by an approach that can be easily extended to other electrocatalytic reactions.

## Graphical abstract

**Moving catalysts on the volcano**: The electrochemical production of hydrogen can be promoted by the synergy between a two‐dimensional polymer and a gold electrode. Engineering the molecular structure of the polymer alters the adsorption energies of the reactants, intermediates, and products and helps to move gold towards the top of the volcano plot.

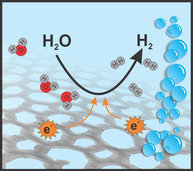

Water electrolyzers, in which H_2_O is split into molecular hydrogen and oxygen, are suitable devices for the storage of intermittently generated excess energy from renewable energy sources such as the sun and wind power.^1^, ^2^ This power‐to‐fuel approach converts electricity into chemical energy and generates an energy‐dense fuel (H_2_) that can be readily stored and transported. On the route towards cheap and efficient electrocatalytic water‐splitting catalysts, the replacement of platinum group metals as catalysts for the hydrogen evolution (HER) half‐cell reaction is a primary milestone.^3^ Whereas platinum and platinum group metals function best under acidic conditions with low pH values,^4^ these highly corrosive conditions inhibit the use of cheaper transition metal based catalysts, and highly acidic proton‐exchange membrane systems are costly and suffer from concerns regarding their stability. More challenging, however, is the lower activity of hydrogen evolution catalysts in alkaline media compared to acidic media.^5^ For equal overpotentials, exchange current densities are commonly lower in an alkaline electrolyte than in an acidic electrolyte, and current efforts are underway to close this gap.^6^, ^7^, ^8^ The rate‐limiting step here seems to be the scission of the HO−H bond in the Volmer step, which precedes the adsorption of hydrogen, and which can be accelerated by designing adequate co‐catalysts. One approach is the deposition of a material that stabilizes H_2_O close to the electrode through hydrogen bonding.^6^, ^9^ The increased interaction results in the faster generation of adsorbed hydrogen, which can then combine to form H_2_ on platinum, effectively leading to a higher HER activity.

Elaborating on this concept, we use molecular engineering to pattern a well‐defined gold electrode surface with an organic polymer with a tunable chemical structure, with the aim of increasing the HER activity at pH 13. The 2D porous single‐layer polymer is structurally similar to the organic sheets that build up 2D covalent organic frameworks (COFs). COFs have recently emerged as active photocatalysts for hydrogen production,^10^, ^11^, ^12^ whereby the organic frameworks can efficiently absorb light as a result of their suitable band gaps. On single‐crystal electrode supports, related single‐layer 2D polymers can be fabricated that pattern the electrode surface with a covalent porous network.^13^, ^14^, ^15^ Through a deliberate choice of the chemical structure of the polymer, we can pattern the gold electrode on the nanoscale with hydrogen‐bonding sites. In this organic‐inorganic hybrid catalyst, the polymer functions as a co‐catalyst that alters the binding strength of the reactants at the gold surface, which ultimately increases the HER activity.

In this comprehensive study, we use scanning tunneling microscopy (STM), X‐ray photoelectron spectroscopy (XPS), electrochemical (EC) measurements, and density functional theory (DFT) calculations to draw a concise picture of the catalyst before and after the HER. Three different brominated precursor molecules with various nitrogen contents were used for on‐surface polymer synthesis (Figure 1 a; **N_0_**=1,3,5‐tris‐(4‐bromophenyl)benzene, **N_3_**=2,4,6‐tris‐(4‐bromophenyl)‐1,3,5‐triazine, and **N_6_**=2,2′,2′′‐(benzene‐1,3,5‐triyl)‐tris‐(5‐bromopyrimidine)). Established Ullmann‐type coupling procedures^16^, ^17^, ^18^, ^19^ on an atomically clean Au(111) substrate allow patterning the surface with a one‐monolayer‐thin porous 2D network (Figure 1 b). For the three precursor molecules **N_0_**, **N_3_**, and **N_6_**, the corresponding covalent 2D polymers **P‐N_0_**, **P‐N_3_**, and **P‐N_6_** exhibit an amorphous network structure with pores of different shapes, mostly hexagons, pentagons, and heptagons. The repeat unit of the polymer consists of two debrominated precursor molecules. **P‐N_0_** is composed purely of carbon and hydrogen, whereas **P‐N_3_** contains triazine heterocycles at the vertices of the network and **P‐N_6_** contains pyrimidine groups in the network's “struts” (see Figure 1 a). All three polymers are physisorbed on the Au(111) surface. The void pores expose the underlying bare substrate and leave it accessible to electrolyte molecules. The polymer‐covered gold substrates were tested for their properties as electrocatalysts, in particular for the HER.

**Figure 1:**
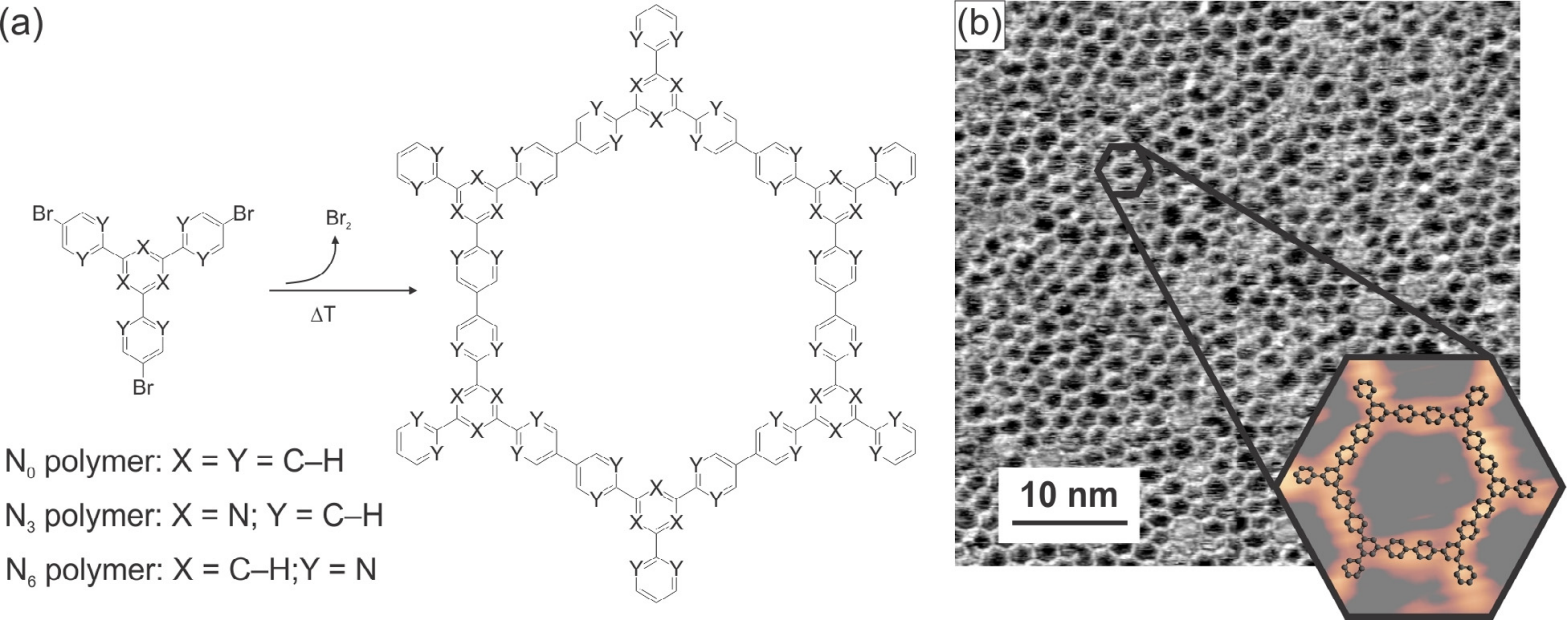
Tunable 2D polymers for electrocatalytic hydrogen evolution: a) Synthesis of the polymers. b) STM topograph (*I*=175pA, *U*=−1.2 V) of **P‐N_0_**. The inset shows an enlargement of a hexagonal pore with a molecular model overlaid.

Cyclic voltammograms of the polymers on the Au(111) surface show characteristic EC signals (Figure 2 a) in the potential window −0.70 to −0.95 V_Ag/AgCl_ which are not present from the bare Au(111) surface (see the Supporting Information for further discussion). The presence of these signals is an electrochemical indicator for the polymers and goes along with an increased HER activity. Polarization curves at more negative potentials of bare Au(111), **P‐N_0_**, **P‐N_3_**, and **P‐N_6_** are shown in Figure 2 b; different current densities for negative potentials are observed as a consequence of the different HER activities. Current densities are depicted with respect to the geometrical area of the crystal; electrochemically active surface areas are equal for all considered systems (see Figure S3 in the Supporting Information). Bare Au(111) shows a current density of −0.16 mA cm^−2^ at a potential of −1.3 V_Ag/AgCl_, while the presence of the **P‐N_3_** increases this value more than threefold to −0.55 mA cm^−2^. **P‐N_0_** and **P‐N_6_** both afford a current density of −0.27 mA cm^−2^, almost twice as large as Au(111). For comparison, the overpotentials versus the reversible hydrogen electrode at −0.2 mA cm^−2^ are **P‐N_3_**: −0.23 V, **P‐N_0_**: −0.29 V, **P‐N_6_**: −0.30 V, and gold: −0.35 V.

**Figure 2:**
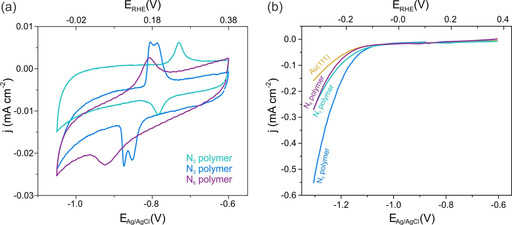
a) Cyclic voltammograms in a 0.1 m Ar‐saturated NaOH solution of **P‐N_0_** (turquoise), **P‐N_3_** (blue), and **P‐N_6_** (purple). b) Polarization curves at 0.05 V s^−1^ in 0.1 m Ar‐saturated NaOH solution for bare Au(111) (yellow), **P‐N_0_** (turquoise), **P‐N_3_** (blue), and **P‐N_6_** (purple).

To ensure that the polymer is present during catalysis and does not decompose prior to or during the HER, we performed X‐ray photoelectron spectroscopy (XPS) of all three polymers before and after catalysis. Exemplarily, Figure 3 shows the characterization of **P‐N_3_** together with typical STM data acquired before and after the HER (three polarization scans up to −1.2 V_Ag/AgCl_; the spectra and images for **P‐N_0_** and **P‐N_6_** are shown in the Supporting Information). No changes are apparent by STM, apart from the filling of the pores with what presumably are residues of the electrolyte (Figure 3 a,b), which is also reflected in the unaltered C 1s core level signal (Figure 3 d). The signal around 284 eV is due to the carbon atoms in the phenyl rings in the polymer, the smaller signal around 286 eV originates from the carbon atoms in the triazine ring. In the N 1s spectrum before the HER, only one component of the triazine nitrogen atoms is observable (Figure 3 c). After the HER, however, additional signals are observed at higher binding energies around 400 eV and 401 eV. These additional signals potentially come from the interaction of the nitrogen atoms with other species such as hydrogen. Literature data confirm that attaching hydrogen to nitrogen shifts the core level to higher binding energies; for example, the signal for protonated amino groups^20^ is shifted from ca. 399.3 eV to ca. 401.2 eV, the iminic nitrogen atom of free‐base porphyrins^21^ is observed at 398.0 eV and the pyrrolic nitrogen atom at 400.1 eV. The hydrogen species attached to the polymer at its nitrogen sites is an intermediate of the HER and its implications for the reaction mechanism will be discussed below. It is noteworthy that exposure of **P‐N_3_** to the electrolyte without applying any potential already leads to the appearance of a peak at 400 eV, indicative of the interaction of water with the polymer (see Figure S8). From this observation we can infer that the nitrogen atoms in the polymer act as interaction sites for water molecules, which stabilize H_2_O close to the gold electrode. The HER activity starts to decrease after extended catalytic turnover (Figure S5), possibly because of degradation of the polymer (see XPS in Figure S7).

**Figure 3:**
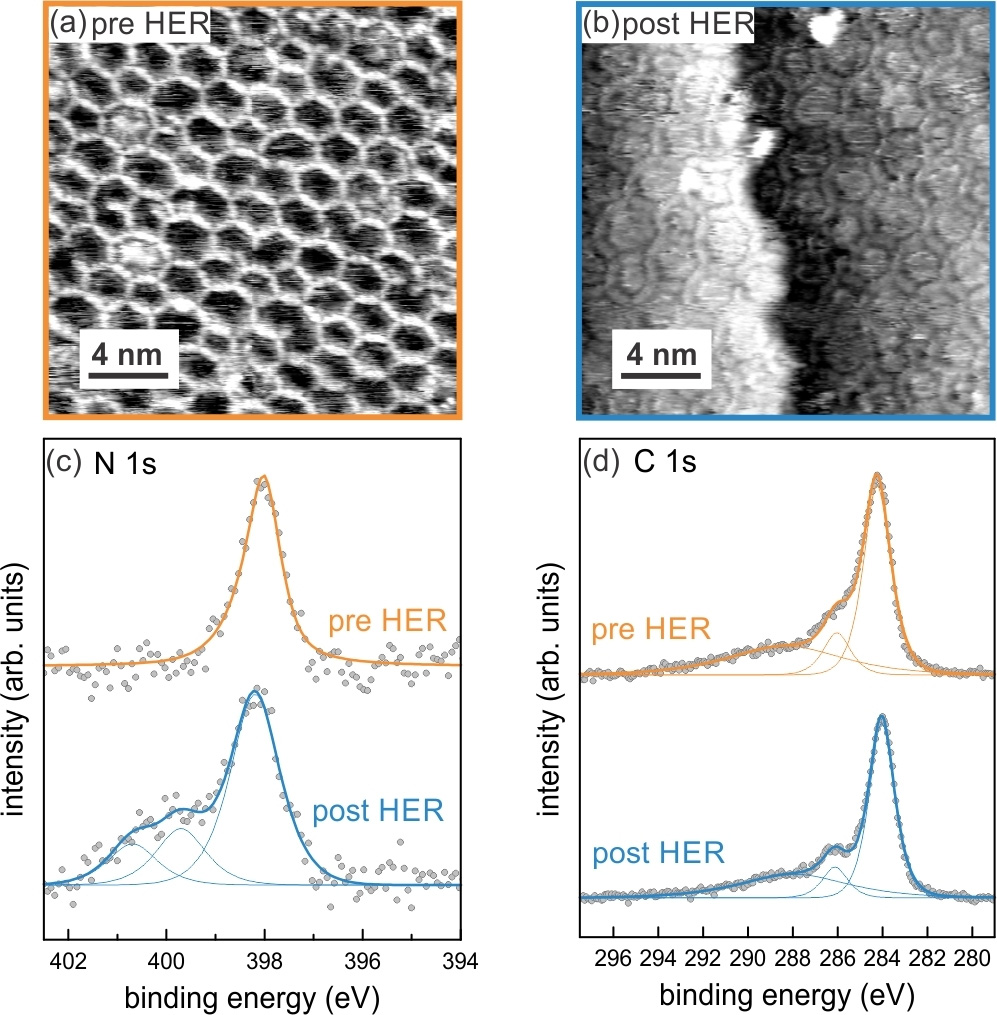
Characterization of **P‐N_3_** before and after the HER: a) STM image before the electrochemical experiment, and b) after the electrochemical experiment. c) XPS spectra of the N 1s core level, orange: before HER, blue: after HER, d) XPS spectra of the C 1s core level, orange: before HER, blue: after HER.

Understanding the observed trend in the HER activity (**P‐N_3_**>**P‐N_6_**≈**P‐N_0_**>Au(111)) calls for the help of theoretical support to deliver insight into the different steps in the conversion of H_2_O into hydrogen molecules. Catalytic activity is usually rationalized in terms of the Sabatier principle, which describes the optimum catalyst such that the interaction of the adsorbed intermediate species is neither too weak nor too strong. If the interaction is too weak, the intermediate does not bind to the catalyst, but if it is too strong, it is too stable and the reaction cannot proceed. The Sabatier principle is graphically illustrated by plotting an observable parameter connected with the catalyst activity (e.g. the electrochemical current in electrocatalysis) as a function of one or more adequate properties that quantify the strength of the interaction of the reaction intermediates with the catalyst (e.g. heats of adsorption). This gives rise to the so‐called volcano plots, with the highest catalytic activity (i.e. the summit of the volcano) being shown by the catalyst characterized by the optimal value of the reaction descriptor(s).

The usual descriptor used for the HER is the hydrogen binding energy (HBE).^22^, ^23^, ^24^ However, since the HBE is an intrinsic property of the catalyst, it is not suitable to account for the effect of external parameters such as the pH value of aqueous electrolytes. Since the electrode surface in an aqueous electrolyte is covered with water, it is likely that the adsorption/desorption of hydrogen is accompanied by desorption/readsorption of water.^25^, ^26^ Thus, a more suitable descriptor proposed recently is the apparent hydrogen binding energy (HBE_app_), which also takes the water binding energy (WBE) into account through HBE_app_=HBE−WBE.^25^ The HBE_app_ descriptor includes pH‐dependency in describing electrocatalytic activities, whereby hydrogen adsorption remains a microscopic reaction step for both acidic and basic electrolytes. Since it was recently shown that the adsorption of hydrogen is the critical reaction step for all pH values,^27^ we rationalize in the following our observed HER activities using HBE_app_ as a descriptor.

As Pt(111) is the benchmark catalyst for the HER, it is expected that the most active catalyst of the investigated systems has an HBE_app_ value close to that of Pt(111). We used DFT to compute HBE, WBE, and HBE_app_ values for **P‐N_3_**, **P‐N_0_**, **P‐N_6_**, and Au(111) as well as for Pt(111). This requires searching for the optimum adsorption geometry of atomic hydrogen and H_2_O in all the investigated systems. We found the optimum adsorption geometry for H_2_O in all investigated polymers at the vertices of the organic network, where water molecules form hydrogen bonds with the polymer, involving the N atoms in **P‐N_6_** (see Figure S9).

For atomic hydrogen on **P‐N_3_** and **P‐N_6_**, the most favorable adsorption site is at the nitrogen atoms, in line with the N 1s XPS data shown above. These results point to N atoms acting as docking sites for both H_2_O and atomic hydrogen. In contrast, the optimal adsorption environment of atomic hydrogen on **P‐N_0_** is the same as on bare Au(111). The preferred adsorption site for atomic hydrogen is at threefold‐hollow sites of Au(111) rather than in proximity to the polymer (Figure S10), which is also in line with the unaltered C 1s XPS signal.

Figure 4 a shows the WBE and HBE_app_ values corresponding to the optimum adsorption geometries found for the three polymer‐decorated surfaces and for Au(111). The HBE_app_ (WBE) values obtained for **P‐N_3_**, **P‐N_6_**, **P‐N_0_**, and Au(111) are +0.02 eV (−0.38 eV), +0.32 eV (−0.77 eV), +0.64 eV (−0.44 eV), and +0.51 eV (−0.31 eV), respectively. The HBE_app_ values in Figure 4 a were calculated as the energy difference between the energies represented in the right gray panel (H adsorption) and in the central panel (H_2_O adsorption). In light of the previous discussion, the activity of each catalyst correlates with its HBE_app_ value and can be compared to that of Pt(111), which is HBE_app,Pt_=−0.07 eV. The measured values of the current densities (j) as a function of the computed values of HBE_app_ are shown in Figure 4 b. The value of HBE_app,Pt_ is indicated with a vertical dashed line. According to the previous analysis, the polymer with the HBE_app_ value closest to Pt is **P‐N_3_**, which is the one with the largest HER activity. We connect the high activity of **P‐N_3_** to an optimized balance between water and hydrogen binding energies that is similar to Pt(111). Compared with **P‐N_3_**, **P‐N_6_** binds H_2_O stronger than atomic hydrogen, which diminishes the electrocatalytic activity, in good agreement with experiments. Hydrogen adsorption on Au(111) becomes endothermic when considering the WBE, thereby inducing a further increase in HBE_app_ and a reduction of its expected catalytic activity with respect to **P‐N_3_** and **P‐N_6_**. Thus, our theoretical results are in line with the experimental trend of HER activities: **P‐N_3_**>**P‐N_6_**>Au(111), although there remains a discrepancy concerning the **P‐N_0_** system. The HBE_app_ descriptor does not account for the measured activity of **P‐N_0_**, which is higher than the activity of Au(111) and similar to that obtained for **P‐N_6_**. This result indicates that other reaction steps might be rate‐delimiting and further parameters are required for a full description of all the investigated systems.

**Figure 4:**
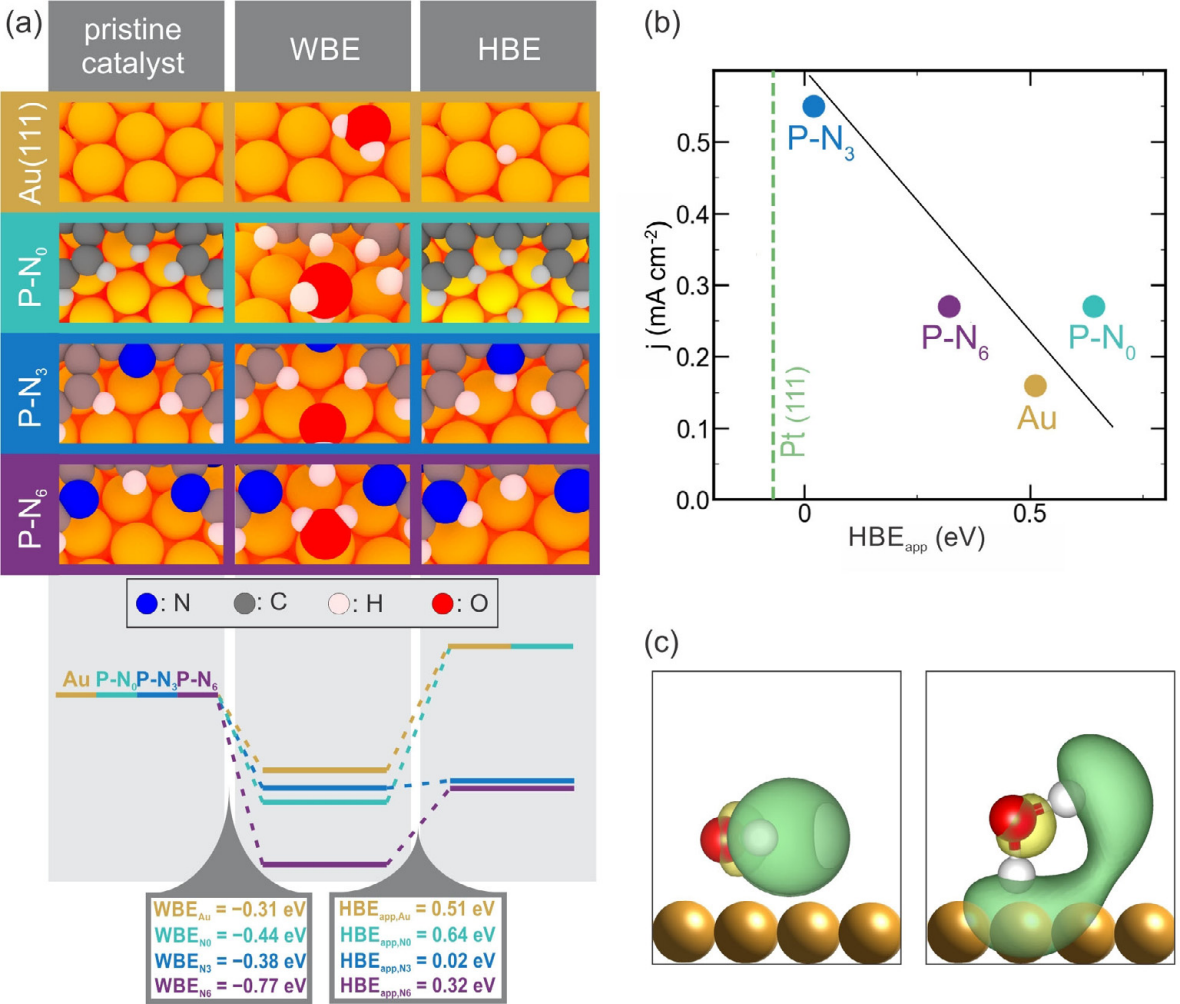
a) Energy diagram for H_2_O and hydrogen adsorption as well as adsorption geometries on Au(111) and on the polymers. b) Plot of HER activity versus the calculated HBE_app_ value, the solid line is to guide the eye. c) Antibonding orbitals of water for parallel and H‐down adsorption configurations.

For example, the activity of the supported **P‐N_0_** and **P‐N_3_** polymers might be reinforced by the particular binding geometries. Our calculations show that single H_2_O molecules adsorb parallel to the surface on apex sites, which is in good agreement with previous theoretical results.^28^ In the case of **P‐N_0_** and **P‐N_3_**, one hydrogen atom of the water molecule points downwards towards the gold electrode (H‐down configuration), while for **P‐N_6_** the H_2_O lies parallel to the surface and forms hydrogen bonds with two N atoms of the polymer. The H‐down geometry of H_2_O (Figure 4 a) favors interaction with the gold electronic states, and consequently electrons transfer into the antibonding 4a_1_ and 2b_2_ orbitals of water (Figure 4 c). As a consequence, this might destabilize the H−O bond, thereby increasing water dissociation and the generation of hydrogen. A similar overlap of the unoccupied antibonding orbitals of water is absent on Au(111) and **P‐N_6_**. We note that a similar H‐down configuration that we propose to facilitate reductive charge transfer into H_2_O is observed in calculations on bare Au(111) and Pt(111).^29^ At room temperature, the water layer on gold becomes disordered, whereas on platinum it remains at least partially in the H‐down configuration; should this adsorption geometry be responsible for the high activity of Pt, then we can expect **P‐N_0_** and also **P‐N_3_** to operate in the same way. Acknowledging that hydrogen adsorption is the relevant descriptor for HER activity, we can thus correlate catalytic activity to the binding energies of H_2_O and atomic hydrogen on the polymer‐decorated surface. Further properties might also influence the details of the reaction, but are probably not the rate‐limiting steps; for example, once H_2_ is formed at a vertex of the polymer, H_2_ abstraction is accompanied by a barrier not larger than about 0.17 eV, which is certainly much lower than all other barriers.

In conclusion, the hydrogen evolution activity of a gold electrode in an alkaline electrolyte was enhanced through patterning the electrode surface with a porous two‐dimensional polymer. The polymer provides docking sites for H_2_O and atomic hydrogen through hydrogen bonds and increases the binding energy and residence time close to the electrode, which consequently increases the catalytic activity. Systematic engineering of the hydrogen bonding sites of the polymer was used to adjust the HER activity. Our results highlight the advantage of controlling the interaction of the reactant with the electrode patterned with porous polymers to achieve hybrid organic/inorganic catalysts: The interaction of the reactants and intermediate products with the electrode can be fine‐tuned by providing adequate docking sites. The binding energy can be dialed‐in such that the optimal energy on the abscissa of the corresponding volcano plot can be achieved for any given metal catalyst just by encoding suitable interaction sites into the precursor molecules. One can envision that different docking sites such as hydroxy groups or sulfur heteroatoms on the polymer would further tweak the hydrogen bonding interactions and position the reactants in a suitable geometry for increased activity. This paves the way for enhancing the catalytic activity not only of the HER but also for other electrocatalytic reactions on earth‐abundant transition‐metal catalysts.

## Conflict of interest

The authors declare no conflict of interest.

## Supporting information

As a service to our authors and readers, this journal provides supporting information supplied by the authors. Such materials are peer reviewed and may be re‐organized for online delivery, but are not copy‐edited or typeset. Technical support issues arising from supporting information (other than missing files) should be addressed to the authors.

Supplementary
