# Supplementary material for: Enhancing Hydrogen Evolution Activity of Au(111) in Alkaline Media through Molecular Engineering of a 2D Polymer
Source: Angew Chem Int Ed Engl. 2020 Mar 18;59(22):8411–5. doi: 10.1002/anie.201915855 (PMC7317855; doi:10.1002/anie.201915855)
Supplement: Supplementary file 1 — Supplementary [file ANIE-59-8411-s001.pdf]

Angewandte Chemie
Eine Zeitschrift der Gesellschaft Deutscher Chemiker

Supporting Information

# Enhancing Hydrogen Evolution Activity of Au(111) in Alkaline Media through Molecular Engineering of a 2D Polymer

*Patrick Alexa, Juan Manuel Lombardi, Paula Abufager, Heriberto Fabio Busnengo, Doris Grumelli, Vijay S. Vyas, Frederik Haase, Bettina V. Lotsch, Rico Gutzler,* and Klaus Kern*

anie_201915855_sm_miscellaneous_information.pdf

## Methods

Prior to polymer synthesis, Au(111) single crystals were cleaned by repeated $Ar^+$-sputtering and annealing (825 K) cycles in ultra-high vacuum (UHV) at a base pressure of < $5\times10^{-10}$ mbar. The porous 2D polymers were synthesized through sublimation of brominated precursor molecules – 1,3,5-tris-(4-bromophenyl)-benzene (**$N_0$**), 2,4,6-tris-(4-bromophenyl)-1,3,5-triazine (**$N_3$**), and 2,2',2''-(benzene-1,3,5-triyl)-tris-(5-bromopyrimidine) (**$N_6$**) – from quartz crucibles and their thermal activation in an Ullmann-like polymerization reaction on the surface. Sublimation temperatures were 475 K for **$N_0$** and 515 K for **$N_3$**, and 515 K for **$N_6$**, while the Au substrate was held at 565 K. The STM images were recorded at room temperature and WsXM[1] was used for image analysis. X-ray photoelectron spectroscopy was performed using a SPECS Phoibos 150 analyzer with a non-monochromatic Mg Kα source (hv = 1253.6 eV). Spectral analysis was performed by fitting Voigt functions of a fixed Lorentzian/Gaussian ratio of 70% after subtraction of the background.
A home-built transfer system was implemented between UHV and the EC cell to ensure contamination free experiments. After sample preparation and visualization of the molecular networks by STM, the sample was moved to the transfer chamber and a pressure of 1 bar Ar atmosphere was established. Before the sample was characterized by STM after electrochemical experiments, the sample surface was rinsed with milliQ water, dried in Ar atmosphere and heated up to 375 K to remove most of residual electrolyte adsorbates. HER experiments were performed in a three-electrode conventional electrochemical cell. 0.1 M NaOH electrolyte was prepared by using NaOH pellets (Sigma-Aldrich, 99.99% trace metals basis) and milliQ water (18.2 MΩ). Ar gas (5.0 purity) was used to purge oxygen from the electrolyte. All potentials are referred to a silver/silver chloride (3 M KCl) reference electrode. Pt coiled wire was used as counter electrode. Linear scans and cyclic voltammetry experiments were performed by using an Autolab potentiostat (Ecochemie Inc, PGSTAT 302N).

**$N_6$** synthesis is described in detail in the Supporting Information and was synthesized for the purpose of testing hypotheses regarding EC data interpretation. Molecule **$N_3$** was synthesized as described in Ref. [2]. **$N_0$** was obtained from commercial sources (Sigma-Aldrich).

Calculations were performed with the VASP[3] code. A plane wave basis set and the projected augmented wave (PAW) method[4] with an energy cut-off of 450 eV was used. Different schemes were tested in order to improve the description of vdW interaction: the PBE+D3[5,6] approach and the optB86, opt88, opt-PBE, DF2 non-local correlation functionals[7,8]. In all cases **$P\text{-}N_x$** hydrogenated monomers absorbed on Au(111) in very similar flat geometry. As a result, the PBE+D3 scheme is used in all further calculations. The surface was modeled by a three-layer fixed slab with unreconstructed Au(111) termination. The lattice is modeled by a rectangular 5×3√3 unit cell with ~15 Å of vacuum. In each case we use one hydrogenated monomer molecule per unit cell. All geometry optimizations were carried out until the forces on every mobile atom were smaller than 0.02 eV $Å^{-1}$. The Brillouin zone sampling was carried out according to the Monkhorst and Pack method with a 3×3×1 mesh. The binding energy for water adsorption WBE is calculated as:

$$WBE = E(H_2O_{ads}/Sys) - E(Sys) - E(H_2O) \qquad \text{(eq.1)}$$

where $E(H_2O_{ads}/Sys)$, $E(Sys)$, and $E(H_2O)$ are the energies of $H_2O$ adsorbed on the system, the system (slab+molecule) itself, and an $H_2O$ molecule in vacuum, respectively.

The binding energy of hydrogen can be expressed as

$$HBE = E(H_{ads}/Sys) – E(Sys) – ½ E(H_2) \qquad \text{(eq. 2)}$$

with $E(H_2)$ the energy of $H_2$ in gas phase.

The apparent binding energy of hydrogen[9], $HBE_{app}$, is calculated as

$$HBE_{app} = HBE – WBE =$$

$$= E(H_{ads}/Sys) – ½ E(H_2) – E(H_2O_{ads}/Sys) + E (H_2O_{gas}) \qquad \text{(eq. 3)}$$

Equation 3 shows that the balance between hydrogen adsorption (HBE) and water adsorption (WBE) energies rules the behavior of $HBE_{app}$ for the different systems.

**Synthesis of 2,2',2''-(benzene-1,3,5-triyl)-tris-(5-bromopyrimidine) ($N_6$):**

1,3,5-Phenyltriboronic acid, tris(pinacol) ester was synthesized by a modified, previously reported method[10]: Anhydrous dioxane (20 ml) was placed in a 20 ml Biotage microwave vial. Subsequently, 1,3,5-tribromo benzene (1.95 mmol, 612 mg ), bis(pinacolato)diboron (7.8 mmol, 1.980 g), potassium acetate (17.54 mmol, 1.721 g) and [1,1'-bis(diphenylphosphino) ferrocene]dichloropalladium(II) (0.12 mmol, 87.8 mg) were added and degassed by bubbling argon through the mixture. The vial was sealed and heated in the microwave under autogenous pressure for 16 h to 90°C. After cooling down the mixture was poured into 50 ml of water and extracted with DCM (3 × 50 ml). The organic fractions were combined and the solvent removed under reduced pressure. The obtained solid was purified by dissolution in boiling hexane (100 ml) and filtering off any insoluble parts while hot. The hexane is then removed and the solid recrystallized from methanol to yield a white crystalline solid (667 mg, 75%). Purity and identity were assessed by $^{1}H$ and $^{13}C$ NMR that matched with the literature reported spectra.[10]

5-bromo-2-iodo Pyrimidine was synthesized from pyrimidinone hydrochloride as previously reported.[11]

2,2',2''-(benzene-1,3,5-triyl)-tris-(5-bromopyrimidine) (**$N_6$**): 30 ml toluene, 10 ml water and 10 ml ethanol were degassed by bubbling argon for 30 min. To this solvent mixture 1,3,5-phenyltriboronic acid, tris(pinacol) ester (2.63 mmol, 1.2 g), 5-bromo-2-iodo pyrimidine (10.53 mmol, 3 g), cesium carbonate (7.9 mmol, 2.6 g), potassium carbonate (7.9 mmol, 1.1 g) and tetrakis(triphenylphosphine)palladium(0) (0.13 mmol, 150 mg) were added. The reaction mixture was first stirred at room temperature for 8 h and then heated to 90°C for 16 h. After cooling down, the reaction mixture was added to 100 ml of water and extracted with 3 × 100 ml DCM. The organic fractions were combined and the solvent removed. The crude product could be purified by either column chromatography in 10% dichloromethane in chloroform. To the product containing fraction, one part ethanol was added and the solvent removed under ambient pressure until precipitation started. Upon cooling to room temperature the solid was filtered off to obtain the pure product. Alternatively, the crude product could be recrystallized from very small amounts of chloroform to obtain an off white powder (749 mg, 52%). $^{1}H$-NMR $CDCl_3$ (300 MHz, ppm): 9.60 ppm (s, 3H), 8.92 ppm (s, 6H); $^{13}C$ NMR (75 MHz, ppm): 162.34, 158.21, 138.05, 130.52, 119.11.

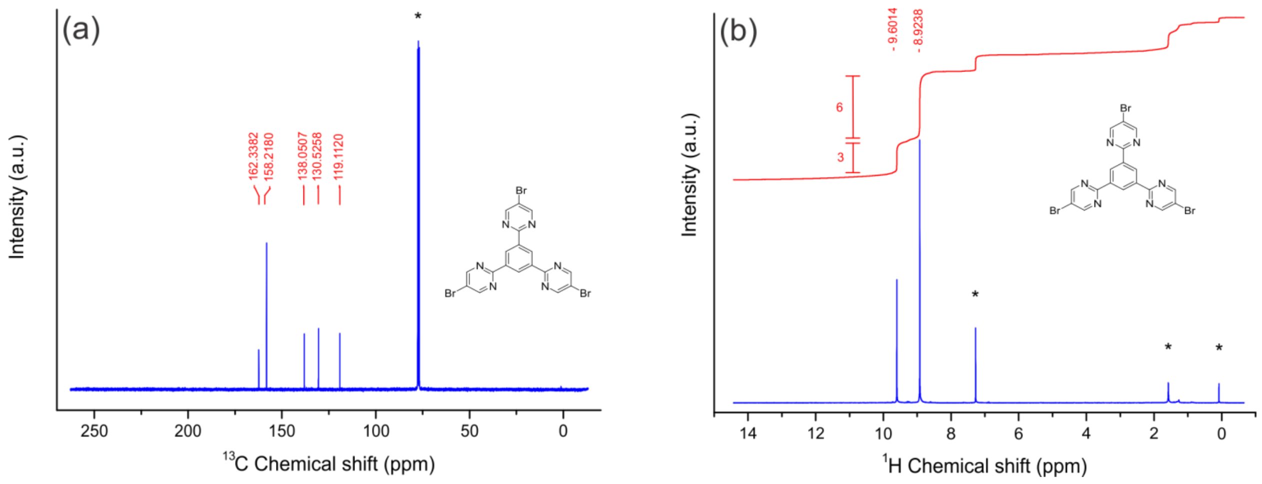


**Figure S1:** NMR spectra of 2,2',2''-(benzene-1,3,5-triyl)-tris-(5-bromopyrimidine) (**$N_6$**)

**Additional electrochemical characterization**

Polymer-characteristic EC signal

The presence of the polymer **P-N$_x$** on the Au(111) surface is be probed electrochemically in the potential window between –0.60 and –1.05 $V_{Ag/AgCl}$. The cathodic peak of **P-N$_0$** is close to –0.8 V $V_{Ag/AgCl}$, the peak of **P-N$_3$** between –0.8 and –0.9 V $V_{Ag/AgCl}$ and the peak of **P-N$_6$** below –0.9 V $V_{Ag/AgCl}$, see Fig. 2a main text. Scan-rate analysis of **P-N$_3$** on the Au(111) reveals a linear relationship between peak height and scan rate (Fig. S2), suggesting the reduction and oxidation of the adsorbed polymer. We note that similar peaks can be expected for bromine adsorption/desorption,[12] but this should occur at larger potentials and the peak height of the anodic adsorption signal should correlate linearly with the square root of the scan rate,[13] whereas we observe a linear dependence on the scan rate. These observations support our hypothesis of a redox process of the adsorbed polymer.

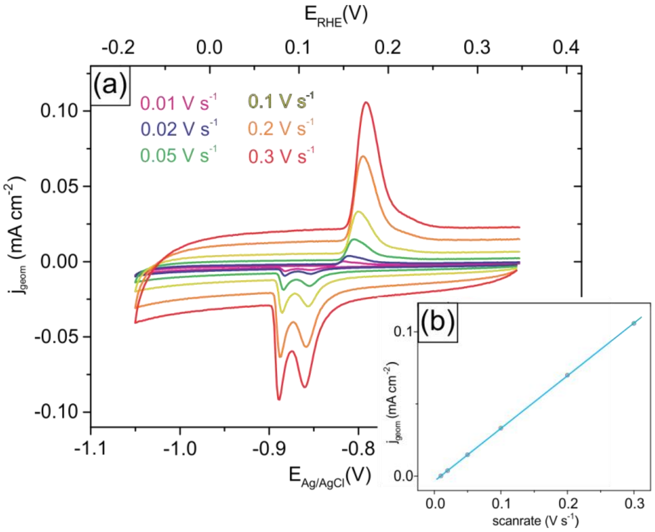


**Figure S2:** (a) Scan-rate analysis of the EC signal. (b) Current density vs. scan rate plot showing a linear relationship.

Electrochemically active surface area (ESCA)

Estimating relative ESCA between different catalysts is desirable if different surface areas of, for example, porous and non-flat catalyst surfaces are being compared.[14] Normalizing the current density to ESCA thus makes comparing catalytic activities possible.[15] For our system, the atomically clean and smooth Au(111) surface that is exposed to the electrolyte is near perfect. One single layer of the porous 2D polymer will not reduce significantly ESCA, as the surface remains flat and most of the gold surface will remain exposed. Figure S3 shows the measured currents as a function of scan rate for Au(111) and

exemplary for **P-N$_3$**. The slope of the linear fit gives the double layer capacitance, which is directly proportional to ESCA.

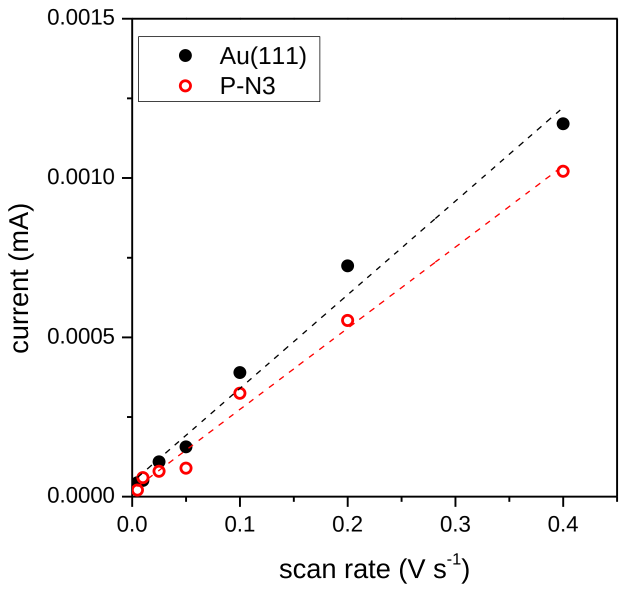


**Figure S3:** Measuring relative electrochemically active surface area for Au(111) and the polymer-decorated surface in 0.1 M Ar saturated NaOH. After determining the open-circuit potential (OCP), a CV is recorded at different scan speed of 0.005, 0.01, 0.025, 0.05, 0.1, 0.2, and 0.4 V $s^{-1}$ within a potential window of ±0.05 V of the OCP. Forward and backward scan currents are averaged; all currents are assumed to be due to capacitive charging in absence of Faradaic processes. The double layer capacitance amounts to 2.9 µF for Au(111) and 2.6 µF for **P-N$_3$**/Au(111). These values are of the same magnitude as previously reported values for gold,[16,17] but placed at the lower end, possibly due to the very smooth electrode surface. The ratio of $C_{P\text{-}N3}/C_{Au(111)} \approx 0.9$ is close to unity and underlines the almost equality of ESCA for pure gold and polymer-decorated surfaces. Since the Au(111) single crystals are perfectly smooth and the deposition of polymers does not alter ESCA, current densities in the main text are reported with respect to the geometrical area.

Ohmic drop

Impedence measurements were carried out to measure the electrolyte resistance in order to estimate the Ohmic drop in our set-up. From impedance measurements we typically get a small electrolyte resistance of 67 Ω (see Fig. S4), which in conjunction with the small currents measured in our experiment does not result in a significant potential drop across the electrolyte (< 10 mV).

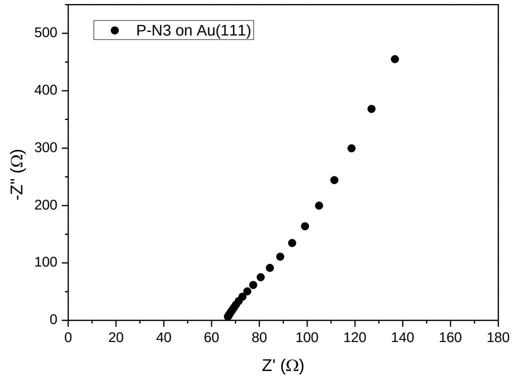


**Figure S4:** Representative Nyquist plot for **P-N$_3$** on Au(111) in 0.1 M Ar saturated NaOH at the open circuit potential, starting frequency at 10000 Hz, voltage modulation 10 mV.

Polymer stability

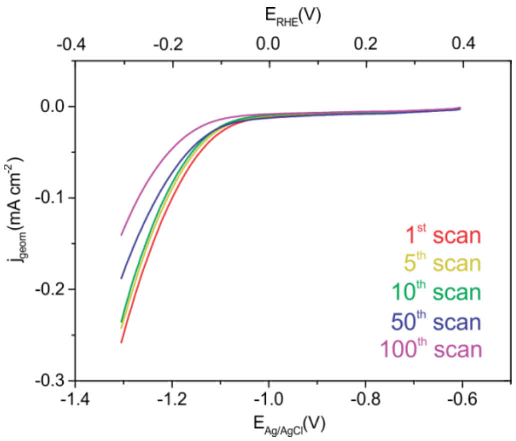


**Figure S5:** HER activity of **P-N$_3$** as a function of number of linear scans. Activity decreases with time spend at large negative potentials. A possible explanation is the limited stability of the polymer under these conditions, which slowly decomposes after extended use for HER. This is in line with XPS data presented below. In addition to a chemical decomposition of the polymer, the polymer might detach from the surface due to the generation of gaseous $H_2$ between polymer and Au(111).

**Additonal STM and XPS data**

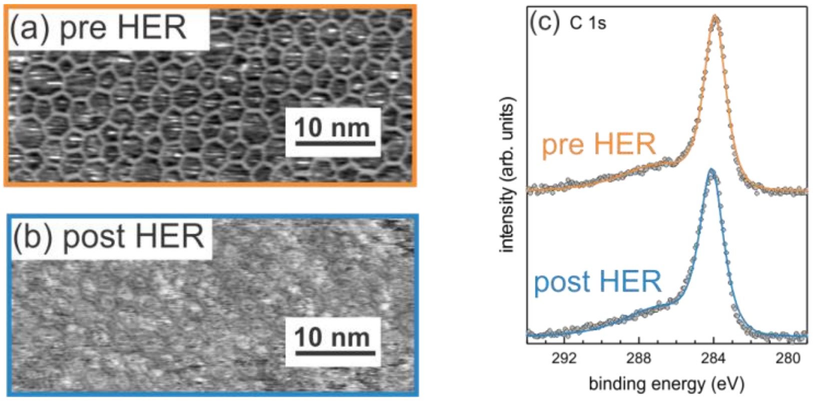


**Figure S6: STM and XPS of P-$N_0$ before and after HER:** (a) STM image recorded before HER and (b) after HER (after 3 LSVs to –1.2 $V_{vs.\ Ag/AgCl}$). Porous polymer structure remains observable after HER. (c) XPS spectra of the C 1s core level, orange: before hydrogen evolution, blue: after hydrogen evolution. No major shifts can be observed indicating the chemical integrity of the polymer

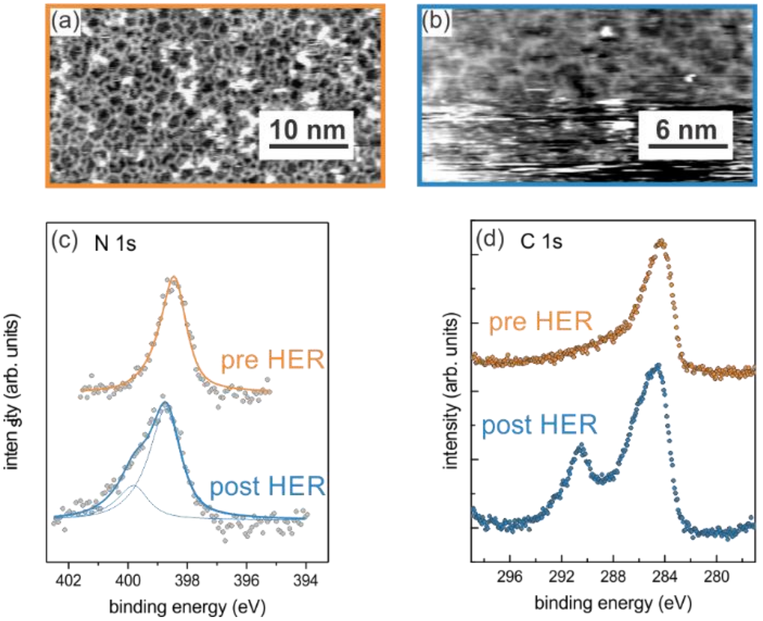


**Figure S7: STM and XPS of P-$N_6$ before and after HER:** (a) STM image recorded before HER and (b) after HER (after 3 LSVs to –1.2 $V_{vs.\ Ag/AgCl}$). Porous polymer structure remains observable after HER. (c) XPS spectra of the N 1s core level and (d) of the C 1s core level, orange: before hydrogen evolution, blue: after

hydrogen evolution. The signal at 398.5 eV corresponds to nitrogen in the pyrimidine groups of **P-N$_6$**. A high binding energy peak in the N 1s spectrum at 399.8 eV shows up after HER and likely stems from nitrogen atoms interacting with the reactants/(intermediate) products/electrolyte, as discussed in the main text for **P-N$_3$**. The additional peak around 290.6 eV in the C 1s spectrum is of unknown origin. We speculate that it originates from hydrolysis of the polymer, which would indicate onset of polymer degradation. The high-binding energy peak can then be related to the presence of electron-withdrawing oxygen in the polymer structure[18].

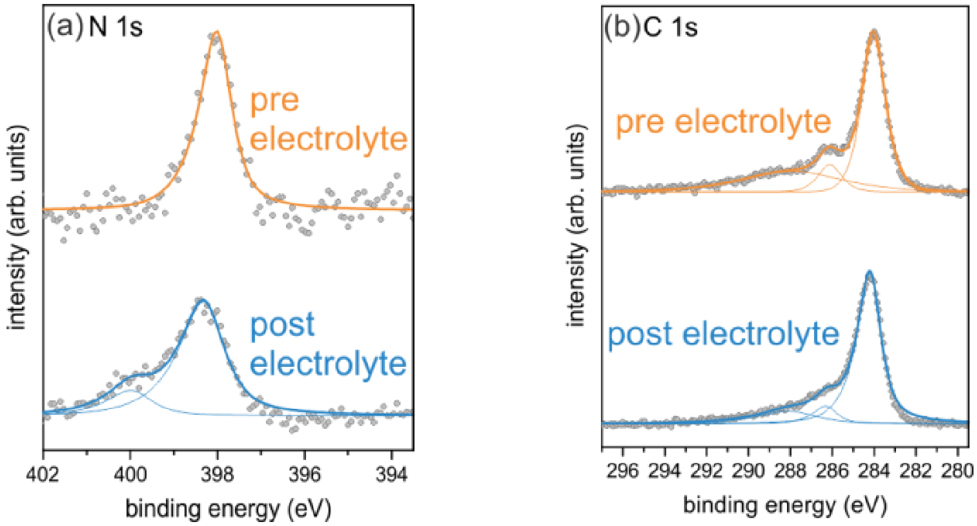


**Figure S8: XPS of P-N$_3$ before and after electrolyte exposure:** The effect of the NaOH electrolyte on the polymer is explored for **P-N$_3$**. The freshly prepared **P-N$_3$** monolayer was exposed to 0.1 M NaOH for 5 minutes. Comparing the N 1s spectrum in (a) before and after electrolyte exposure exhibits the appearance of a second peak at high binding energy in addition to the signal from the triazine nitrogen. This new peak is located at 400.0 eV, which stems from nitrogen atoms interacting with the water from the electrolyte (see main text for discussion). The C 1s spectrum in (b) shows the expected signal, which does not undergo any significant changes upon electrolyte exposure.

**Additonal DFT data**

Figure S9 and Table S1 show the different explored configurations for $H_2O$ adsorption on (a) Au(111), (b) P-$N_6$, (c) P-$N_0$ and (d) P-$N_3$, respectively. In addition, Figure S10 and Table S2 exhibit the different analyzed configurations for H adsorption on (a) Au(111), (b) P-$N_6$, (c) P-$N_0$ and (d) P-$N_3$, respectively. All these exploratory calculations were conducted with a two-layer slab and a Γ-point in the Brillouin zone sampling.

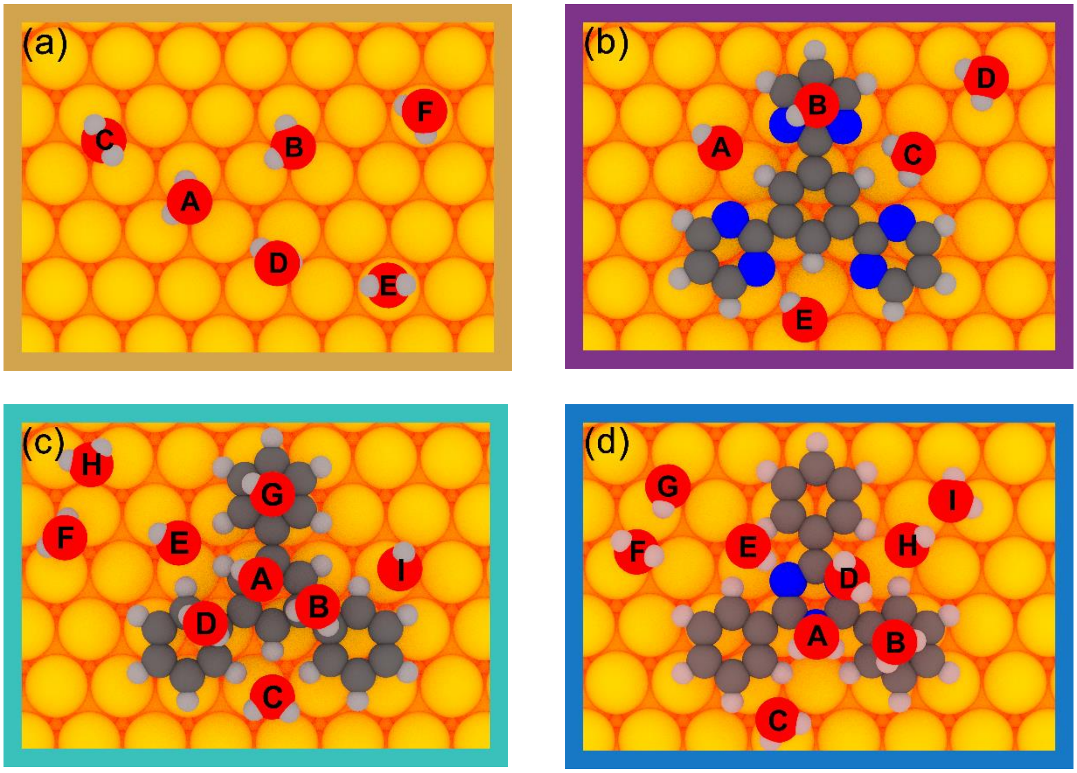


**Figure S9: Explored configurations for $H_2O$ adsorption on the different systems:** (a) Au(111), (b) P-$N_6$, (c) P-$N_0$ and (d) P-$N_3$.

| | Conf. A | Conf. B | Conf. C | Conf. D | Conf. E | Conf. F | Conf. G | Conf. H | Conf. I |
|---|---|---|---|---|---|---|---|---|---|
| Au | -0.30 | -0.29 | -0.07 | -0.28 | -0.07 | -0.29 | - | - | - |
| P-$N_3$ | -0.15 | -0.15 | -0.31 | -0.19 | -0.32 | -0.07 | -0.21 | -0.41 | -0.29 |
| P-$N_0$ | -0.15 | -0.24 | -0.47 | -0.16 | -0.48 | -0.31 | -0.20 | -0.29 | -0.33 |
| P-$N_6$ | -0.37 | -0.17 | -0.74 | -0.31 | -0.33 | - | - | - | - |

**Table S1:** $H_2O$ adsorption energies (WBE) on Au, P-$N_6$, P-$N_0$ and P-$N_3$

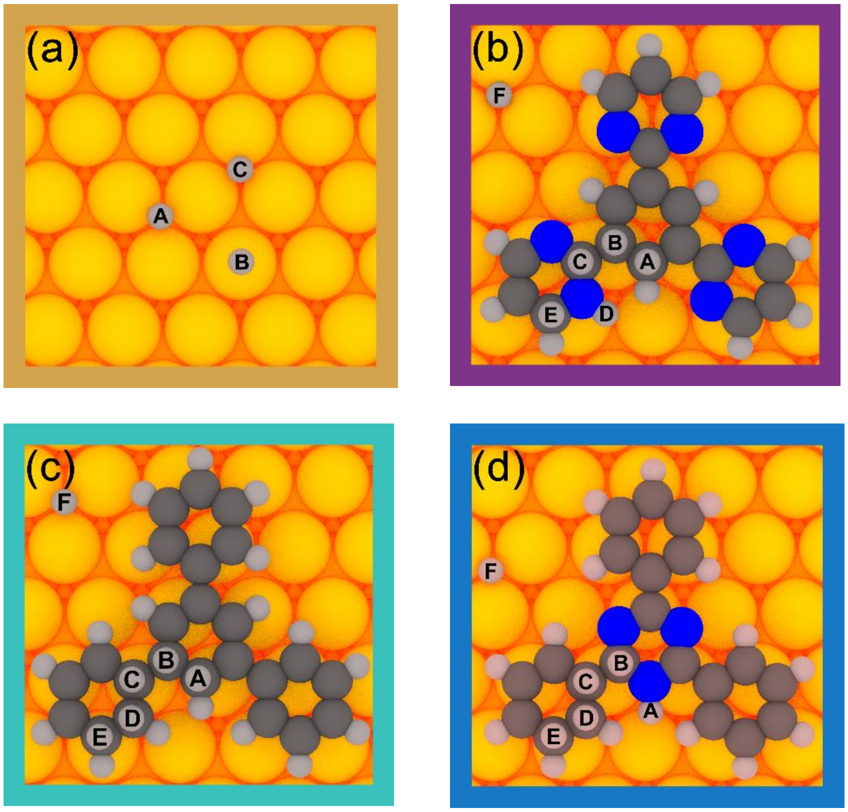


**Figure S10: Explored configurations for H adsorption on the different systems:** (a) Au(111), (b) P-$N_6$, (c) P-$N_0$ and (d) P-$N_3$.

| | Conf. A | Conf. B | Conf. C | Conf. D | Conf. E | Conf. F |
|---|---|---|---|---|---|---|
| Au | 0.075 | 0.245 | 0.075 | - | - | |
| P-$N_3$ | -0.44 | 0.94 | 0.91 | 0.74 | 0.69 | 0.075 |
| P-$N_0$ | 0.22 | 0.97 | 0.92 | 0.27 | 0.69 | 0.075 |
| P-$N_6$ | 0.30 | 1.07 | 0.88 | -0.52 | 0.77 | 0.075 |

**Table S2:** Hydrogen adsorption energies (HBE) on Au, P-$N_6$, P-$N_0$ and P-$N_3$

For lower energy structures of H and $H_2O$ adsorption on each system, we used a 3×3×1 k-mesh and a three-layer slab. Figure S11 shows HER experimental activity as a function of different potential descriptors for the reaction. In Fig. S11(a) the abscissa is the hydrogen binding energy HBE, in (b) the water adsorption energy WBE and in (c) apparent hydrogen binding energy $HBE_{app}$.

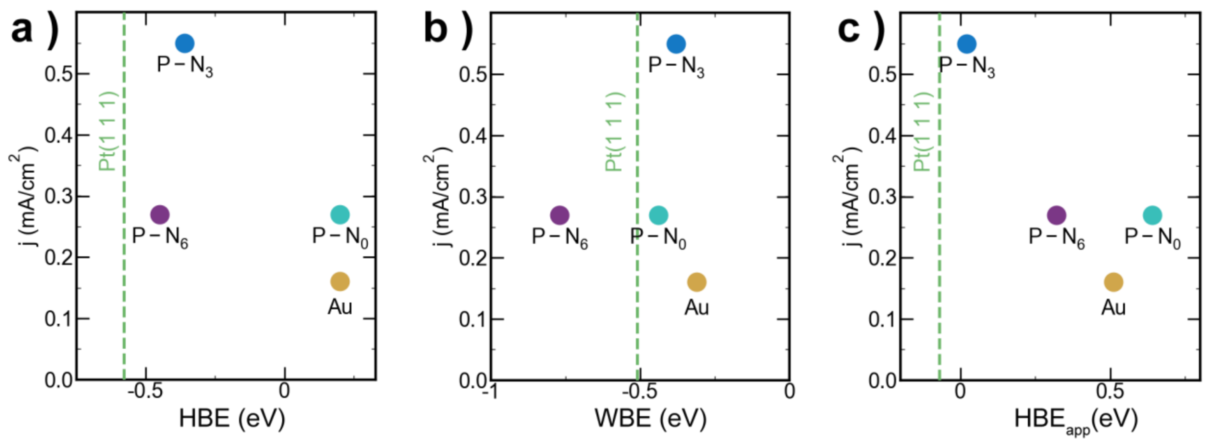


**Figure S11: HER experimental activity as a function of different potential descriptors for the reaction:** (a) hydrogen binding energy HBE, (b) water adsorption energy WBE and (c) apparent hydrogen binding energy $HBE_{app}$.

[1] I. Horcas, R. Fernández, J. M. Gómez-Rodríguez, J. Colchero, J. Gómez-Herrero, A. M. Baro, *Rev. Sci. Instrum.* **2007**, *78*, 013705.

[2] V. S. Vyas, F. Haase, L. Stegbauer, G. Savasci, F. Podjaski, C. Ochsenfeld, B. V. Lotsch, *Nat. Commun.* **2015**, *6*, 8508.

[3] G. Kresse, J. Furthmüller, *Comput. Mater. Sci.* **1996**, *6*, 15–50.

[4] G. Kresse, D. Joubert, *Phys. Rev. B* **1999**, *59*, 1758–1775.

[5] J. P. Perdew, K. Burke, M. Ernzerhof, *Phys. Rev. Lett.* **1996**, *77*, 3865–3868.

[6] S. Grimme, J. Antony, S. Ehrlich, H. Krieg, *J. Chem. Phys.* **2010**, *132*, 154104.

[7] J. Klimeš, D. R. Bowler, A. Michaelides, *J. Phys. Condens. Matter* **2010**, *22*, 022201.

[8] J. Klimeš, D. R. Bowler, A. Michaelides, *Phys. Rev. B* **2011**, *83*, 195131.

[9] J. Zheng, J. Nash, B. Xu, Y. Yan, *J. Electrochem. Soc.* **2018**, *165*, H27–H29.

[10] W. A. Braunecker, K. E. Hurst, K. G. Ray, Z. R. Owczarczyk, M. B. Martinez, N. Leick, A. Keuhlen, A. Sellinger, J. C. Johnson, *Cryst. Growth Des.* **2018**, *18*, 4160–4166.

[11] S. Hug, L. Stegbauer, H. Oh, M. Hirscher, B. V. Lotsch, *Chem. Mater.* **2015**, *27*, 8001–8010.

[12] N. J. Tao, S. M. Lindsay, *J. Phys. Chem.* **1992**, *96*, 5213–5217

[13] M. R. Rezwan, T. Ohsaka, *Electrochim. Acta* **2009**, *54*, 1570–1577

[14] S. Trasatti, O. A. Petrii, *Pure & Appl. Chem.* **1991**, *63*, 711–734

[15] C. C. L. McCrory, S. Jung. J. C. Peters, T. F. Jaramillo, *J. Am. Chem. Soc.* **2013**, *135*, 16977–16987

[16] A. Toyota, N. Nakashima, T. Sagara, *J. Electroanal. Chem.* **2004**, *565*, 335–342

[17] M. B. Cortie, A. I. Maaroof, G. B. Smith, *Gold Bulletin* **2005**, *38*, 14–22

[18] J.-B. Lhoest, P. Bertrand, L. T. Weng, J.-L. Dewez, *Macromolecules* **1995**, *28*, 4631–4637.
